# Abstract of Address Delivered at Opening of Course of Lectures, Rush Medical College

**Published:** 1879-11

**Authors:** C. T. Parkes

**Affiliations:** Professor of Anatomy


### ARTICLE V.

### ABSTRACT OF ADDRESS DELIVERED AT THE OPENING OF THE COURSE OF LECTURES IN RUSH MEDICAL COLLEGE. By C. T. PARKES, M.D., Professor of Anatomy.

Best known among the surgeons of Guy's Hospital, is the author of the popular treatise on surgery, Mr. Thomas Bryant. Mr. Bryant is the most ready and finished lecturer on clinical surgery in London. He is a graduate of the school in which he is the most noted surgeon, is a man of very methodical habits and a prolific writer, keeping up his early practice of making a written abstract of every case which presents anything new or interesting in its history. No man can present more trustworthy statistics in support of any proposition adduced, or more readily recall precedents to justify any course of procedure adopted.

It was my pleasure to listen to the masterly address of Mr. W. S. Savory, one of the surgeons of St. Bartholomew's Hospital, the subject being "Antiseptic Surgery." He was fortunate enough to stir up an enthusiasm which is seldom witnessed in British assemblies. The responses which he succeeded in eliciting did no more than justice to his classical oratory, as well as to the exceedingly interesting and commendable subject matter of the address.

Much of the success thereof was undoubtedly due to the manner of the man himself. Polished, suave, elegant in action and speech, all his attributes show through his quiet simplicity and earnestness, absolutely free from the least particle of demonstrativeness or display. His smooth, clear face exhibits striking lineaments and they speak with such straightforward honesty and impressive thoughtfulness, the fullest measure of the listener's confidence is instantly secured and retained. You feel that every sentence uttered by his lips comes backed up with the support of thoughtful consideration and wisely elaborated experience, while the story is told so plainly, so free from all subterfuge or disingenuousness, that conviction of its trustworthiness is forced upon you. Again, antiseptic surgery was a subject to gain everybody's ear; treating, as it does, of principles in the care of wounds, which have excited more attention than any heretofore presented to the consideration of medical men.

The claim was made that as good results could be obtained by the judicious use of rest, cleanliness and abundance of fresh air, in the treatment of injuries, as ever followed the use of Prof. Lister's method of carbolization; further, it was claimed that the theory of exclusion of elements of fermentation in such treatment, did not reach the necessities of all cases, that it was harmful to some; and, that there was much in the condition of the wound itself sufficient to work bad influence, against which the gauze furnished no protection.

That these hurtful accompaniments were best avoided by adopting means to solicit union by first intention, by the free use of pure water and by the establishment of easy drainage by position to prevent auto-infection.

The claims were supported by clear arguments and wonderfully excellent hospital statistics. This was not based upon a trial of Mr. Lister's method, for that was practically ignored. There was no comparison of methods of treatment but merely an enunciation of the simplest plans of procedure that can possibly be adopted in the treatment of wounds, with a statement of the grand results worked out of the means used.

It was no attack on Mr. Lister; only an exposition of another way — certainly much easier and claimed to be as safe — to reach the great aim for which both men have been striving — the saving of human life and prevention of human suffering. No man, no assemblage of men, can ever deprive Mr. Lister of the just fame ever to be associated with his name in repayment for the elucidation and introduction of principles of treatment which have revolutionized surgical practice and so widely extended its field of relief and usefulness.

My own view, gentlemen, is that one objection to the gauze dressings lies in the disturbance of wounds during the re-application of them. The opinion is hazarded that if Mr. Savory would make use of Mr. Lister's antiseptic principles and dressings, and so modify their application as to allow to the fullest extent the employment of the very valuable principle of undisturbed rest, then his statistics would show still greater victories over the ravages of septic poisoning. * * * *

St. Bartholomew's Hospital was founded in 1123 by one Rayhere, a minstrel to Henry I. He made his money and obtained his possessions by singing songs to "my lords and ladies." In later life he took up the role of Prior, and among many other ways of doing good, established this home for the sick. In 1544 Henry VIII re-endowed and re-established it, being "moved thereto with great pity for, and towards the relief and succor and help of the poor, aged, sick, low and hurt people, lying and going about begging in the common streets of the city of London and the suburbs of the same, and infected with divers great and horrible sicknesses and diseases."

It is the richest and oldest of London's hospitals — the best patronized both by patients for its beds and pupils in its college.

Although many changes have certainly been made in the buildings during the years of its existence, still its walls must be reeking with bacteria, the fermenting seeds left by the hosts of patients who have received care and attention during its lifetime, in spite of its outward show of extreme cleanliness. Surely it is a justifiable pride which boasts of the success of treatment carried out within its wards, and speaks in tones of the highest commendation of the worthy men who have had supervision of them and the care of their inmates.

One of the surgeons of this hospital, a genial, kindly gentleman — of whose practice I saw a great deal — is Mr. Geo. W. Callender. He is a brilliant operator, a zealous student and a most earnest and practical advocate of the benefits of *rest.* For, it *is* providing for *rest* to dress a limb so that no quivering muscles shall pull asunder the delicate bonds holding together freshly wounded surfaces — it *is* providing for *rest* so to plan any necessary incisions as to secure a dependent opening, to obtain ready drainage for all waste fluids and preventing the harmful separation of coapted flaps, and avoiding the danger of the dire consequences resulting from absorption by open-mouthed vessels. Not the least of his brilliant attributes is his kindly nature and full-hearted sympathy, which keeps alive his devotion to these all-powerful of nature's indications, causing him to be unceasing in his vigilance to bring comfort and ease to every suffering patient who falls into his hands.

It is hardly necessary to say anything of the adjuncts, purity and abundance of air and of absolute cleanliness. Their necessity is so self-evident that no words are required to impress the importance of their usefulness or the dreadful consequences sure to follow their absence or even scanty provision. So, here in this best and oldest of hospitals, it was my privilege to see exemplified and put into practice, with results grand beyond comparison, the very principles so ably, earnestly and repeatedly insisted upon by an honored preceptor and respected colleague within the walls of my own Alma Mater. It was among the most delightful experiences I ever enjoyed. It made me alive to the knowledge which was really my own, but which I had never fully appreciated or developed. Knowledge of any kind, no matter how long owned or possessed, is but a cumbersome adornment, unless put to use by practical application.

---

THE French are afraid that the invention of their countryman, M. Guillotin, for the extermination of criminals, may be supplanted by that of an American named Packard, who has devised a box, in which the criminal is rapidly asphyxiated by carbonic oxide gas.

